# Molecular Mechanisms and Clinical Evidence Supporting the Four Pillars of Therapy in Diabetic Kidney Disease: Emerging Therapeutic Perspectives

**DOI:** 10.3390/ijms27062757

**Published:** 2026-03-18

**Authors:** Hidekatsu Yanai, Hiroki Adachi, Mariko Hakoshima, Hisayuki Katsuyama

**Affiliations:** Department of Diabetes, Endocrinology and Metabolism, National Kohnodai Medical Center, Japan Institute for Health Security, 1-7-1 Kohnodai, Ichikawa 272-8516, Chiba, Japan; adachi.h@jihs.go.jp (H.A.); hakoshima.m@jihs.go.jp (M.H.); katsuyama.h@jihs.go.jp (H.K.)

**Keywords:** diabetic kidney disease, dotinurad, glucagon-like peptide-1 receptor agonists, imeglimin, nonsteroidal mineralocorticoid receptor antagonists, pemafibrate, sodium-glucose cotransporter-2 inhibitors

## Abstract

Diabetic kidney disease (DKD) is one of the most serious complications of diabetes and the leading cause of end-stage renal disease worldwide. Recently, renin-angiotensin system inhibitors, non-steroidal mineralocorticoid receptor antagonists, sodium-glucose cotransporter 2 inhibitors, and glucagon-like peptide-1 receptor agonists were proposed as the four pillars for treating DKD. To understand the molecular mechanisms by which these drugs improve DKD, we described the histological and molecular changes due to diabetes. Based on our understanding of the molecular changes in DKD, we present evidence on the efficacy of these drugs in improving DKD and discuss why such drugs improve the prognosis of DKD. In addition to diabetes and hypertension, insulin resistance, dyslipidemia and hyperuricemia are risk factors for DKD. Metformin, fibrates, and febuxostat have been reported to improve DKD; however, caution is required when administering these drugs to patients with renal impairment due to concerns about the onset of lactic acidosis, rhabdomyolysis, and deterioration of renal function, respectively. Imeglimin, pemafibrate, and dotinurad have similar chemical structures or effects to metformin, fibrates, and febuxostat, respectively, but are safer in patients with renal impairment. Furthermore, they have specific mechanisms to improve DKD and may offer new options for its treatment. This article is a narrative review. Regarding emerging therapies for DKD, no high-evidence-level research has yet been published, and further progress in this area is warranted.

## 1. Introduction

Diabetic kidney disease (DKD) is defined by albuminuria and progressive reduction in estimated glomerular filtration rate (eGFR) in the setting of a long duration of diabetes [1]. The diagnosis of DKD is most conclusively made by findings of mesangial expansion and nodular glomerulosclerosis on kidney biopsy, though kidney biopsy is rarely necessary. DKD is one of the most serious complications of diabetes and the leading cause of end-stage renal disease (ESRD) worldwide [2]. DKD is widespread, causing serious health problems and imposing a significant economic burden on human societies worldwide [3].

Risk factors of DKD include hyperglycemia, hypertension, dyslipidemia, and obesity/insulin resistance [4]. Hyperglycemia is considered one of the most prominent and independent risk factors for DKD, as it worsens renal function by altering the antioxidant system, leading to an increased formation of advanced glycation end products (AGEs) [5]. Hyperglycemia also activates the polyol pathway, enhancing the development of DKD [5]. Randomized controlled trials (RCTs), such as the ACCORD trial and VADT trial, have demonstrated the beneficial effects of intensive glucose control on the delayed onset and prevention of albuminuria progression in patients with type 2 diabetes [6,7].

Hypertension is also a pivotal risk factor for DKD. A meta-analysis including 27 studies showed an increased risk of DKD with hypertension by 67% compared to without hypertension [8]. In the prospective cohort study of 158,365 Chinese men and women, compared with those with normal blood pressure, prehypertension and stage 1 and stage 2 hypertension increased the development of ESRD by 30%, 47% and 106%, respectively [9].

Dyslipidemia plays an important role in the development and progression of DKD. A recent meta-analysis comprising 165,230 studies found that low high-density lipoprotein cholesterol (HDL-C) levels (<40 mg/dL) were associated with a 70% increase in DKD incidence [10]. Another recent meta-analysis demonstrated that low-density lipoprotein-cholesterol (LDL-C) was significantly higher in DKD patients who developed ESRD [11].

Obesity/insulin resistance is a critical risk factor for type 2 diabetes, hypertension, and dyslipidemia; therefore, obesity is closely related to the development of DKD. In the meta-analysis, abdominal obesity was significantly associated with an increased odds of DKD [12]. A recent meta-analysis showed that high body mass index (BMI) was a risk factor for adverse kidney events such as the onset of DKD, the doubling of serum creatinine, ESRD, or death in patients [13].

In diabetes, the renin-angiotensin system (RAS) is activated, especially in the kidneys and heart, driven by hyperglycemia and insulin resistance, leading to increased angiotensin II, which causes inflammation, oxidative stress, fibrosis, and further insulin resistance, thereby driving diabetic complications such as nephropathy and cardiovascular disease [14]. RAS is also implicated in the pathogenesis of primary hypertension [15]. Obesity activates the RAS through multiple pathways, including increased sympathetic nervous system activity, adipose tissue inflammation, insulin resistance, and direct effects of adipokines such as leptin, leading to higher blood pressure and sodium retention [16]. Activated RAS is significantly associated with the development of DKD, which may explain why RAS inhibitors (RASis) and non-steroidal mineralocorticoid receptor antagonists (nsMRAs) have improved renal outcomes in DKD.

Recently, RASis, nsMRAs, sodium-glucose cotransporter 2 inhibitors (SGLT2is), and glucagon-like peptide-1 receptor agonists (GLP-1RAs) have been proposed as the four pillars for treating DKD [17]. These drugs have demonstrated a significant prevention of DKD progression via different mechanisms in RCTs.

However, how these drugs affect the pathology of DKD and how they improve it have not been fully elucidated. We conducted a literature search to clarify the mechanisms by which these drugs improve DKD. Furthermore, we conducted an extensive literature search, including animal studies, to identify drugs that may improve the pathology of DKD, even though these have not been proven in high-level evidence studies, such as RCTs.

## 2. Methods

This article is a narrative review. We used PubMed to obtain information on the pathology of DKD and its therapies. We included peer-reviewed English-language articles and excluded all other articles. To search for information on risk factors and the histological and molecular characteristics of DKD, we used the keywords “DKD and risk factors” and “DKD and glomerulus”, “DKD and mesangial cells”, “DKD and podocyte”, “DKD and endothelial cell”, “DKD and arteriolosclerosis”, “DKD and interstitial fibrosis”, “DKD and proximal and distal tubular damage”, “DKD and collecting duct”, “DKD and RAS”, and “DKD and MR”, respectively, without a timeframe of literature inclusion. To gather information on the mechanism of action of each drug for DKD, we searched the literature using keywords for each drug, combined with the above-listed terms, without a timeframe for inclusion. However, to collect information on risk factors, the histological and molecular characteristics of DKD, and the mechanism of action of each drug for DKD, we included as many recent articles as possible.

We used the articles that form the basis of the four pillars of DKD, which were those published in high-impact-factor journals and conducted as RCTs with a high level of evidence, such as the RENAAL Study (2001) for RASis, the FIDELIO-DKD study (2020) for nsMRA, the EMPA-REG OUTCOME trial (2016) for SGLT2i, and the LEADER Trial (2017) for GLP-1RA. To explore emerging therapies for DKD, we investigated the effects of new drugs targeting risk factors for DKD developed after the EMPA-REG OUTCOME trial (2016), such as dotinurad for hyperuricemia, imeglimin for type 2 diabetes, and pemafibrate for dyslipidemia.

## 3. Results and Discussion

### 3.1. The Histological and Molecular Characteristics of DKD

**Figure 1:**
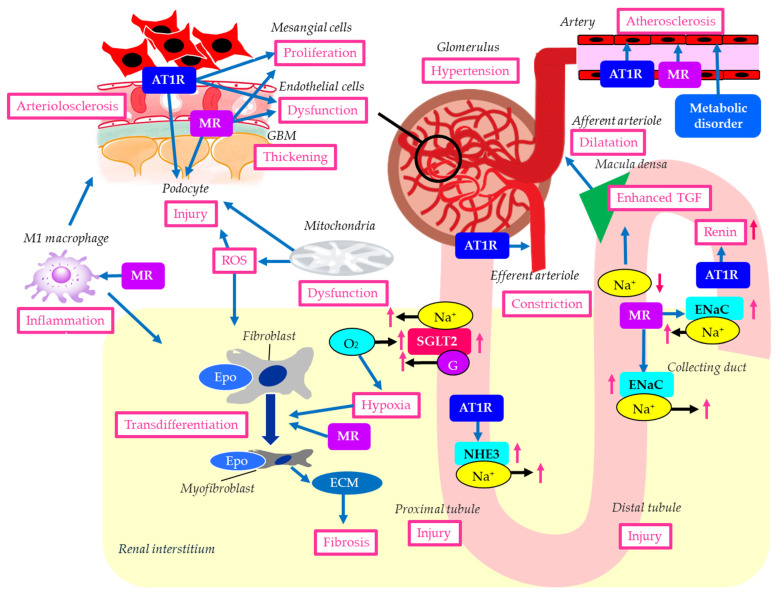
The histological and molecular characteristics of DKD. Blue arrows indicate effects, black horizontal arrows indicate the flow of substance, and red upward and downward arrows indicate an increase and a decrease of activity or substances, respectively. AT1R, angiotensin II type 1 receptor; ECM, extracellular matrix; ENaC, epithelial sodium channel; Epo, erythropoietin; G, glucose; GBM, glomerular basement membrane; MR, mineralocorticoid receptor; NHE3, Na+/H+ exchanger 3; ROS, reactive oxygen species; SGLT2, sodium-glucose cotransporter 2; TGF, tubuloglomerular feedback.

The histological and molecular characteristics of DKD are illustrated in Figure 1. Histological findings of DKD include proliferation of mesangial cells, thickening of the glomerular basement membrane (GBM), podocyte damage, glomerular endothelial dysfunction and arteriolosclerosis, interstitial fibrosis, and proximal and distal tubular damage [18].

Glomerular hyperfiltration contributes to the development of DKD. Hyperglycemia induces the dilatation of afferent arterioles through the release of vasoactive mediators, including insulin-like growth factor 1 (IGF-1), glucagon, nitric oxide (NO), vascular endothelial growth factor (VEGF), and prostaglandins [19]. Due to a high filtered load of glucose, reabsorption of glucose and sodium is increased because of the upregulation of SGLT2 in the proximal tubules. Reduced delivery of sodium to the macula densa of distal tubules causes dilatation of the afferent arteriole because of enhanced tubuloglomerular feedback (TGF) [20]. At the same time, constriction of the efferent arteriole occurs due to a high local level of angiotensin II, which causes glomerular hypertension [21].

Erythropoietin levels increase after initiation of SGLT2is treatment in diabetic patients. In patients with diabetes, the proximal tubules are overloaded with excessive glucose reabsorption, and the increased oxygen demand leads to tubulointerstitial hypoxia [22]. Consequently, erythropoietin production is impaired because of the dysfunction of fibroblasts. SGLT2is reduce the workload of the proximal tubules and improve tubulointerstitial hypoxia, allowing fibroblasts to resume normal erythropoietin production.

A study investigating the effect of arteriosclerosis on new-onset renal damage in a Chinese community population with diabetes demonstrated that atherosclerosis is a risk factor for new-onset renal damage, particularly new-onset proteinuria, in patients with diabetes [23]. A cross-sectional study revealed that vascular inflammation, atherosclerosis, and altered lipid metabolism were associated with the development of DKD [24].

Hyperglycemia, hypertension, and angiotensin II are considered the primary factors contributing to mesangial cell proliferation [25]. Podocytes play a crucial role in maintaining the integrity of the glomerular filtration barrier [26]. Hyperglycemia induces oxidative stress, inflammation, and mitochondrial damage, thereby leading to podocyte injury [27]. Hypertension, dyslipidemia, and vascular endothelial cell damage also contribute to podocyte injury and subsequent loss of glomerular filtration barrier integrity [28]. Emerging evidence suggests that mitochondrial dysfunction is a key driver of podocyte injury in DKD [24]. Impairment of mitochondrial function results in an energy crisis, oxidative stress, inflammation, and cell death [28].

Renal interstitial fibrosis, caused by hyperglycemia and inflammation, is characterized by the accumulation of the extracellular matrix (ECM) and the transdifferentiation of renal tubular epithelial cells and fibroblasts into myofibroblasts, ultimately leading to excessive ECM production. Transforming growth factor beta (TGF-β) promotes this cellular transformation and ECM deposition, leading to renal fibrosis [29]. Hyperglycemia, dyslipidemia, oxidative stress, hypoxia, activation of RAS, endoplasmic reticulum (ER) stress, inflammation, epithelial–mesenchymal transition, and programmed cell death have been shown to induce renal tubular injury [30].

In the kidney with DKD, the infiltrating macrophages were mainly M1 macrophages [31]. In DKD, crosstalk between the Notch pathway and nuclear factor-κB (NF-κB) signaling in macrophages contributes to macrophage polarization. Hyperpolarized macrophages secrete great amounts of inflammatory cytokines, exacerbating the inflammatory response, ECM secretion, fibrosis, and necroptosis in glomerular mesangial cells and renal tubular epithelial cells [31].

Diabetes was associated with a 3.5-fold increase in collecting duct renin production via the angiotensin II type 1 receptor (AT1R) [32]. When DKD occurs, it stimulates the RAS, one of the most significant indicators of developing renal impairment. Intrarenal RAS components are upregulated in patients with DKD, contributing to its development [33]. Increased urinary renin could contribute to RAS activation, which is relevant to sodium retention, hypertension, glomerular hemodynamics, inflammation, and the progression of DKD. Elevated urinary renin levels were found in patients with DKD [34]. The AT1R in the proximal tubule plays a crucial role in regulating blood pressure and fluid balance by controlling sodium (Na+) reabsorption, primarily through the Na+/H+ exchanger 3 (NHE3). Angiotensin II activates this receptor to increase Na+ uptake [35].

In diabetes, AT1Rs in the glomerulus and renal tubules are overactivated by hyperglycemia, inducing DKD by increasing oxidative stress, inflammation, and ECM deposition [36]. Angiotensin II binds to AT1Rs on mesangial cells, podocytes, and tubules. AT1R activation induces mesangial cell proliferation and stimulates them to produce excess ECM, impairing filtration. Hyperglycemia and angiotensin II lead to the overproduction of reactive oxygen species (ROS) and inflammatory signals, ultimately damaging podocytes [36].

In diabetes, angiotensin II binding to AT1R on endothelial cells promotes vascular damage by increasing ROS, reducing nitric oxide (NO) production, inducing inflammation, and causing cellular senescence and endothelial-to-mesenchymal transition [37]. AT1Rs are found on the vascular smooth muscle cells of both the afferent and efferent arterioles. Angiotensin II, acting primarily through AT1R, constricts both the afferent and efferent renal arterioles, a crucial mechanism for regulating GFR and systemic blood pressure [38]. Glomerular pressure is maintained by the balance in tone between the afferent and efferent arterioles. Glomerular hyperfiltration is an early manifestation of DKD and most likely results from dysregulation of afferent and efferent arteriolar tone [39]. Angiotensin II can cause relative vasoconstriction of the efferent arteriole, increase glomerular pressure, and lead to hyperfiltration [40].

MR in the kidney, primarily activated by aldosterone, regulates salt/water balance in the distal tubules, which is crucial for maintaining blood pressure. MR activation contributes to the propagation of kidney injury, inflammation, and fibrosis, as well as the progression of chronic kidney disease (CKD) [41]. MR can be activated by nonligand activation with the regulatory protein Rac family small guanosine triphosphatase 1 (Rac1), oxidative stress, elevated glucose, or high salt levels [42]. Rac1 mediates MR activation, and constitutively active Rac1 has been shown to induce nuclear translocation and activation of MR in podocytes [43]. Mice with elevated Rac1 activity developed podocyte injury and severe albuminuria, which were prevented by MR antagonism [43,44]. MR overactivation in podocytes may induce podocyte injury.

MR is expressed in mesangial cells, where its activation by aldosterone promotes inflammation, oxidative stress, fibrosis, and excessive cell proliferation through pathways such as the extracellular signal-regulated kinase pathway [45].

Autophagy is an essential process for podocyte maintenance and is disrupted in DKD [46]. Spironolactone improved DKD by promoting podocyte autophagy [47]. Aldosterone mediates podocyte injury through the NLRP3 inflammasome, a member of the NOD-like receptor family, and pyrin domain-containing 3 (NLRP3) [48]. Furthermore, aldosterone induces podocyte injury by reducing podocyte-associated molecules and activating the Wnt/β-catenin signaling pathway [49].

Endothelial dysfunction is reported in patients with CKD, which may be mediated by the endothelial MR [50]. Aldosterone increases the expression of cell adhesion molecules and monocyte chemoattractant protein-1 (MCP-1) in endothelial cells [51]. Aldosterone also promotes oxidative injury [52,53] and reduces endothelial nitric oxide synthase (eNOS) activity [54].

Aldosterone activates MR in the distal tubule and collecting duct to increase sodium reabsorption via the epithelial sodium channel (ENaC), thereby increasing blood pressure [55]. It has been shown that ENaC activation increases salt sensitivity in patients with diabetes [56,57]. Increased sodium retention enhances intraglomerular pressure that could lead to podocyte injury, mesangial expansion, and kidney fibrosis. Thus, regulating ENaC to stimulate sodium excretion would be an important therapeutic target for preventing DKD progression.

In myeloid cells, aldosterone stimulates the expression of tumor necrosis factor-α (TNF-α), interleukin-12, and MCP-1 [58]. Infiltration of M1 proinflammatory macrophages promotes maladaptive repair and perpetuation of injury, whereas an early switch from M1 to M2 macrophages promotes effective repair [59]. Mice lacking MR in the myeloid lineage exhibit increased expression of M2 polarization markers in infiltrating macrophages [60].

Increased MR activation may lead to fibroblast proliferation and the production of profibrotic molecules, inducing renal fibrosis [61]. Aldosterone stimulates ECM synthesis [62], but platelet-derived growth factor-induced ECM production has been shown to be reduced in fibroblasts cultured with spironolactone [63], indicating a significant contribution of MR activation to renal fibrosis.

The endothelial cell MR is a central regulator of vascular dysfunction [64]. The endothelial cell MR disrupts vasomotor control via ENaC activation, eNOS dysregulation, and glycocalyx injury, amplifies inflammation, oxidative stress, and mitochondrial ROS, and drives fibrosis and remodeling via TGF-β [64]. The endothelial cell MR contributes to diabetes- and obesity-related vascular disease.

### 3.2. The Four Pillars for Treating DKD

#### 3.2.1. RASis for DKD

RASis such as angiotensin-converting enzyme inhibitors (ACEis) and AT1R blockers (ARBs) reduce systemic blood pressure by vasodilation. RASis-induced renal vasodilation results in an increase in renal blood flow, leading to improvement of renal ischemia and hypoxia. ARBs reduce urinary albumin by lowering intraglomerular pressure, thereby protecting the glomerular endothelium and podocytes [65]. In addition to blocking angiotensin II-induced renal tissue injury, ARBs can decrease intrarenal angiotensin II levels by reducing proximal tubular angiotensinogen and renin production in the collecting duct, thereby reducing intrarenal RAS activation. As shown in Figure 1, ATR1 activation has been reported to have adverse effects across various kidney regions; therefore, ARBs are thought to improve DKD pathology in multiple ways.

In the RENAAL Study, ARB (losartan) reduced the incidence of doubling of serum creatinine concentration by 25% (*p* = 0.006) and ESRD by 28% (*p* = 0.002) compared with placebo in patients with DKD [66]. The level of proteinuria declined by 35% with losartan (*p* < 0.001 vs. placebo).

Another ARB, irbesartan, reduced the risk of a doubling of the serum creatinine concentration by 33% (*p* = 0.003 vs. placebo) and by 37% (*p* < 0.001 vs. amlodipine) in patients with DKD [67]. Treatment with irbesartan reduced the relative risk of ESRD by 23% compared with placebo and amlodipine (*p* = 0.07 vs. placebo and amlodipine). Serum creatinine increased more slowly in the irbesartan group than in the placebo and amlodipine group. Unlike the RENAAL Study, this study compared ARBs not only with placebo but also with a calcium blocker and showed that ARBs improve renal outcomes independently of blood pressure reduction.

#### 3.2.2. nsMRA for DKD

Although a meta-analysis showed a 31% reduction in urinary protein or albumin excretion after treatment with a steroidal MRA in patients with CKD, data on hard clinical outcomes are lacking [68]. Finerenone, a nonsteroidal, selective MRA, had more potent anti-fibrotic effects than steroidal MRAs in preclinical models [69]. Finerenone has been shown to reduce the urinary albumin-to-creatinine ratio (UACR) in patients with CKD treated with an RASis [70].

Finerenone reduced the risk of the primary composite outcome, including renal failure, a sustained decrease of at least 40% in eGFR from baseline, or death from renal causes, in patients with CKD and type 2 diabetes treated with RASis by 18% in the FIDELIO-DKD study [71]. In this study, the early reduction in albuminuria, the early separation of the Kaplan-Meier curves for the secondary outcome (death from cardiovascular causes, nonfatal myocardial infarction, nonfatal stroke, or hospitalization for heart failure), and the modest reduction in blood pressure suggested that some of the benefits of finerenone may be mediated by its natriuretic effect. Overall, hyperkalemia-related adverse events were twice as frequent with finerenone as with placebo (18.3% and 9.0%, respectively), and the frequency of hyperkalemia leading to discontinuation of the trial regimen was also higher with finerenone (2.3% and 0.9% in the respective groups). However, no fatal hyperkalemia adverse events were reported.

In the RCT, to determine whether the direct renin inhibitor aliskiren would reduce cardiovascular and renal events in patients with type 2 diabetes and CKD treated with RASis, systolic and diastolic blood pressures were lower with aliskiren, and the mean reduction in UACR was greater in the aliskiren group [72]. Although aliskiren demonstrated significant hemodynamic effects in this trial, it did not reduce the risk of the secondary outcome, suggesting that the addition of aliskiren with RASis in patients with type 2 diabetes who are at high risk for cardiovascular and renal events was not supported by this study. Another RCT showed that combination therapy with an ACEi and an ARB was associated with an increased risk of adverse events among DKD patients [73]. These results, along with the FIDELIO-DKD study, highlight the significance of selective MR blockade beyond strong RAS inhibition for the secondary outcome.

Aldosterone breakthrough is a well-known phenomenon that occurs in patients with long-term use of RASis [74]. The blockade of MR is effective in managing patients with resistant hypertension, defined as uncontrollable blood pressure despite the concurrent use of three antihypertensive drugs [74]. Aldosterone breakthrough puts hypertensive patients at a higher risk of cardiovascular disease and worsens future outcomes.

As shown in Figure 1, MR activation has been reported to contribute to deleterious effects in various parts of the kidney through mechanisms distinct from ATR1 activation. Therefore, nsMRAs are thought to ameliorate DKD pathology through multiple mechanisms, not just RAS inhibition. The delayed separation of the Kaplan–Meier curves for the primary outcome and for persistent benefit over the trial duration provides evidence supporting the hypothesis that finerenone may slow CKD progression by influencing tissue remodeling.

#### 3.2.3. SGLT2is for DKD

In the EMPA-REG OUTCOME trial, SGLT2i empagliflozin slowed the progression of kidney disease and reduced the rate of clinically relevant renal events compared with placebo when added to standard care in patients with type 2 diabetes [75]. In this study, during long-term administration, the eGFR remained stable in the empagliflozin groups and declined steadily in the placebo group. The percentages of patients who had an adverse event, a serious adverse event, or an adverse event leading to study-drug discontinuation were similar in the empagliflozin and placebo groups. Genital infections were reported in a higher percentage of patients in the empagliflozin group than in the placebo group. The percentages of patients with confirmed hypoglycemic episodes, diabetic ketoacidosis, thromboembolic events, bone fractures, and events consistent with volume depletion were similar in the two study groups.

In the CANVAS Program, another SGLT2i, canagliflozin, showed a possible benefit with respect to the progression of albuminuria (hazard ratio [HR], 0.73; 95% confidence interval [CI], 0.67 to 0.79) and the composite outcome of a sustained 40% reduction in eGFR, the need for renal replacement therapy, or death from renal causes (HR, 0.60; 95% CI, 0.47 to 0.77) [76]. In the EMPA-REG OUTCOME trial, more than 99% of the patients had established cardiovascular disease; however, in the CANVAS Program, 65.6% of patients had a history of cardiovascular disease.

In patients with DKD, the risk of renal failure and cardiovascular events was lower in the canagliflozin group than in the placebo group at a median follow-up of 2.62 years, in the CREDENCE trial [77]. In the CREDENCE trial, all patients had an eGFR of 30 to <90 mL/min/1.73 m^2^ and albuminuria (UACR > 300 to 5000) and were treated with renin-angiotensin system blockade. The CREDENCE trial included patients with more advanced DKD than the EMPA-REG OUTCOME trial and the CANVAS Program.

The DAPA-CKD trial showed that the risk of a composite endpoint, including a sustained decline in eGFR of at least 50%, ESRD, or death from renal or cardiovascular causes, was significantly lower with dapagliflozin than with placebo in patients with CKD, regardless of diabetes status [78]. In the EMPA-KIDNEY trial, empagliflozin therapy led to a lower risk of progression of kidney disease or death from cardiovascular causes than placebo in a wide range of patients with CKD, regardless of the presence or absence of diabetes [79]. All participants in the EMPA-REG OUTCOME trial, the CANVAS Program, and the CREDENCE trial had type 2 diabetes; however, 67.5% had received a diagnosis of type 2 diabetes in the DAPA-CKD trial, and 54.0% did not have diabetes in the EMPA-KIDNEY trial.

**Figure 2:**
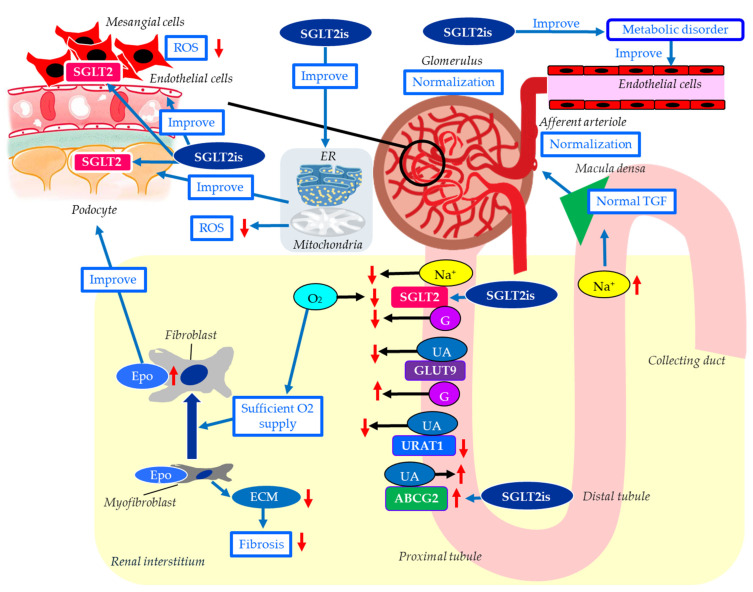
The mechanisms of possible renal protective effects of SGLT2is. Blue arrows indicate effects, black horizontal arrows indicate the flow of substance, and red upward and downward arrows indicate an increase or decrease in activity or substances, respectively. ATP-binding cassette, subfamily G, 2 (ABCG2); ECM, extracellular matrix; Epo, erythropoietin; ER, endoplasmic reticulum; G, glucose; GLUT9, glucose transporter 9; ROS, reactive oxygen species; SGLT2, sodium-glucose cotransporter 2; SGLT2is, sodium-glucose cotransporter 2 inhibitors; TGF, tubuloglomerular feedback; UA, uric acid; URAT1, urate transporter 1.

The mechanisms for possible renal protective effects of SGLT2is were shown in Figure 2. The main mechanism of the renal protective effect of SGLT2is is the sodium-related physiological effects of SGLT2is [20]. Increased SGLT2 mRNA expression increases the renal NaCl reabsorption in the proximal tubule, leading to a marked reduction in distal NaCl delivery to the macula densa [80]. The decline in macula densa NaCl delivery is sensed by the juxtaglomerular apparatus as a reduction in circulating plasma volume, a phenomenon called TGF, leading to maladaptive glomerular afferent arterial vasodilatation and increased intraglomerular pressure [81]. SGLT2is increase distal renal NaCl delivery, inducing an increased afferent tone, thereby reducing the intraglomerular pressure and glomerular hyperfiltration [82].

SGLT2is reduce the overload of the proximal tubules and improve tubulointerstitial hypoxia, which leads to the recovery of erythropoietin production by fibroblasts [22]. Increased hematocrit during SGLT2i therapy indicates the recovery of tubulointerstitial function in diabetic kidneys [22]. In fact, serum erythropoietin increased from baseline in the dapagliflozin group through week 4 [83]. Elevated erythropoietin may contribute to the renal protective effect of SGLT2is [84]. The treatment with recombinant human erythropoietin showed renal protective effects beyond hematopoiesis in streptozotocin-induced diabetic rats [85]. Erythropoietin has been reported to protect podocytes from AGE-induced damage [86]. Erythropoietin ameliorated podocyte injury in advanced diabetic nephropathy in db/db mice [87].

Hyperuricemia may be associated with a higher risk of DKD progression in individuals with type 2 diabetes [88]. Hyperuricemia results from increased purine production coupled with reduced renal or intestinal excretion of uric acid (UA) [89]. In humans, the reabsorption of UA into the blood plays a crucial role in regulating serum UA levels. Renal UA reabsorption is mainly mediated by urate transporter 1 (URAT1) and glucose transporter 9 (GLUT9) expressed in the proximal tubule [90,91,92]. ATP-binding cassette, subfamily G, 2 (ABCG2) has been identified as a high-capacity UA exporter that mediates renal and/or intestinal UA excretion [93]. In a meta-analysis using 59 RCTs, all SGLT2is significantly decreased serum UA levels compared with the placebo [94]. Another meta-analysis showed that SGLT2is significantly reduced the risk of gout in individuals with type 2 diabetes and heart failure [95]. SGLT2is increase urinary glucose and sodium by blocking SGLT2 in the proximal tubule; then, glucosuria and natriuresis augment tubular flow, diluting luminal UA and downregulating URAT1, which reduces UA reabsorption [96,97,98,99]. SGLT2is increase urinary glucose, and increased urinary glucose may compete with urinary UA for apical GLUT9 in the proximal tubules, thereby reducing UA reabsorption [100]. SGLT2is have been reported to upregulate ABCG2, thus promoting urinary UA excretion in diabetic mice [101]. The serum UA-lowering or increased urinary UA excretion effects of SGLT2is may contribute to its renal protective effect.

Exposure of mesangial cells to high glucose levels significantly increased SGLT2 expression [102]. Canagliflozin inhibited high-glucose-induced activation of the protein kinase C (PKC)-NAD(P)H oxidase pathway and increased ROS production [102]. Inhibition of mesangial SGLT2 may reduce PKC activation and ROS production in DKD, contributing to the renoprotective effects of SGLT2 inhibitors.

Recently, SGLT2 has been reported to be expressed in podocytes, and SGLT2is have been shown to improve podocyte damage in kidney diseases [103,104,105]. Empagliflozin showed structural and functional benefits on podocytes of BTBR ob/ob mice [106].

Mitochondria-associated ER membranes (MAMs) are dynamic contact sites between ER and mitochondria that play an important role in the exchange of metabolic substances and messengers between the two organelles and in modulating calcium and lipid homeostasis, apoptosis, inflammation, ER stress, mitochondrial dynamics, and bioenergetics [107]. MAM dysfunction may be a crucial pathogenesis of podocyte damage in DKD [108,109]. SGLT2is have been previously reported to protect mitochondrial and ER functions [110,111]. Inhibition of SGLT2 attenuates MAMs imbalance in diabetic podocytes by activating the AMP-activated protein kinase (AMPK) pathway [112].

Endothelial dysfunction is associated with the development of DKD, and advanced diabetic glomerulopathy often exhibits thrombotic microangiopathy, including glomerular capillary microaneurysms and mesangiolysis, which are typical manifestations of endothelial dysfunction in the glomerulus [113]. Diabetic mice with severe endothelial dysfunction owing to eNOS deficiency develop progressive nephropathy, indicating a significant role of endothelial dysfunction in DKD. SGLT2is have been reported to improve vascular endothelial dysfunction, including improving eNOS [114].

#### 3.2.4. GLP-1RAs for DKD

In the LEADER Trial, the renal outcome occurred in fewer participants in the liraglutide group than in the placebo group (HR, 0.78; 95% CI, 0.67 to 0.92; *p* = 0.003). The new onset of persistent macroalbuminuria occurred in fewer participants in the liraglutide group than in the placebo group (HR, 0.74; 95% CI, 0.60 to 0.91; *p* = 0.004) [115]. New-onset microalbuminuria occurred in fewer patients in the liraglutide group than in the placebo group (HR, 0.87; 95% CI, 0.83 to 0.93; *p* < 0.001). The rates of renal adverse events were similar in the liraglutide group and the placebo group.

In the FLOW Trial, the risk of a primary-outcome event was 24% lower in the semaglutide group than in the placebo group (HR, 0.76; 95% CI, 0.66 to 0.88; *p* = 0.0003) [116]. The mean annual eGFR slope was less steep in the semaglutide group (*p* < 0.001). Serious adverse events were reported in fewer participants in the semaglutide group than in the placebo group (49.6% vs. 53.8%). Adverse events leading to permanent discontinuation of semaglutide or placebo were more common in the semaglutide group than in the placebo group (13.2% vs. 11.9%); this difference was driven mainly by gastrointestinal disorders (4.5% vs. 1.1%).

The GLP-1RA used in the LEADER Trial is a formulation to be injected once daily, while the GLP-1RA used in the FLOW Trial is a formulation to be injected once weekly. Furthermore, while the LEADER Trial targeted patients with type 2 diabetes, the FLOW Trial targeted patients with type 2 diabetes and CKD.

**Figure 3:**
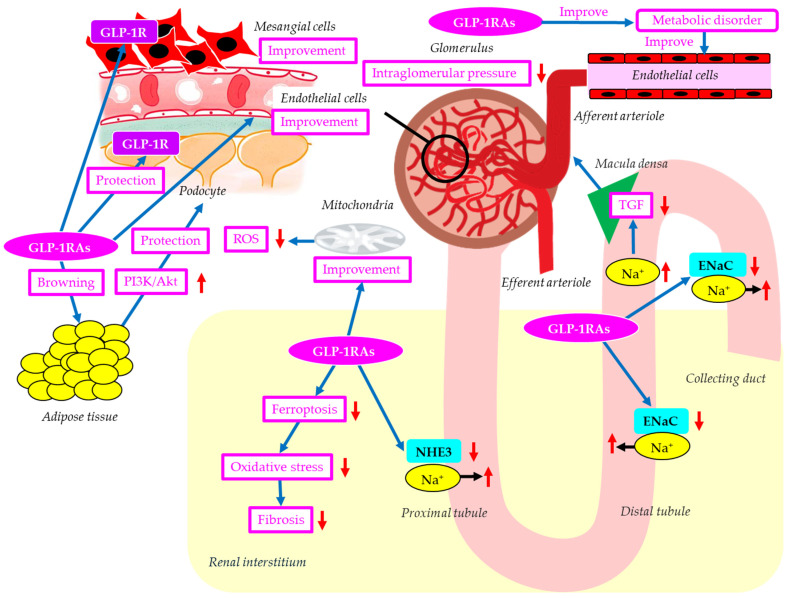
The mechanisms of possible renal protective effects of GLP-1RAs. Blue arrows indicate effects, black horizontal arrows indicate the flow of substance, and red upward and downward arrows indicate an increase or decrease in activity and substances, respectively. ENaC, epithelial sodium channel; GLP-1R, glucagon-like peptide-1 receptor; GLP-1RAs; glucagon-like peptide-1 receptor agonists; NHE3, Na+/H+ exchanger 3; PI3K-Akt, phosphatidylinositol 3′-kinase-Akt; ROS, reactive oxygen species; TGF, tubuloglomerular feedback.

The mechanisms of possible renal protective effects of GLP-1RAs are shown in Figure 3. The phosphatidylinositol 3′-kinase-Akt (PI3K-Akt) signaling pathway plays an important role in podocyte apoptosis [117,118]. GLP-1RAs inhibit podocyte apoptosis by upregulating the PI3K/Akt pathway by promoting white fat browning [119]. GLP-1RAs reversed diabetes- and high-glucose-induced decreases in podocyte GLP-1R expression, suggesting that GLP-1RAs may directly affect podocytes via GLP-1R [119].

Ferroptosis is a regulated mechanism of cellular demise distinguished by iron-dependent oxidative stress and lipid peroxidation [120]. Oxidative stress can damage tubular and vascular endothelial cells and induce the secretion of profibrotic factors, ultimately leading to fibroblast activation and fibroblast/macrophage recruitment [121]. GLP-1RAs attenuated renal fibrosis injury in db/db mice by inhibiting ferroptosis [122].

The natriuretic effect of GLP-1RAs has been proposed to underlie the GLP-1RAs–induced reduction in blood pressure [123]. GLP-1RAs increase natriuresis by reducing NHE3 activity in the proximal tubule [124]. Elevated salt sensitivity by ENaC overexpression may be a risk factor for DKD. GLP-1RAs could ameliorate salt sensitivity by decreasing ENaC expression and help prevent kidney injury in DKD [125]. The increased natriuresis induced by inhibition of NHE3 and ENaC by GLP-1RAs may increase Na+ delivery to macula densa, reducing TGF and resulting in alleviation of glomerular hypertension.

Lipotoxicity, the excessive accumulation of lipids in non-adipose tissues due to abnormal lipid metabolism, is associated with the progression of DKD by inducing ER stress, mitochondrial dysfunction, impaired autophagy, inflammation, and oxidative stress [126]. GLP-1RAs may attenuate lipotoxicity by improving insulin resistance in adipose tissue, enhancing fatty acid (FA) oxidation, reducing FA synthesis, and improving mitochondrial function [127]. Mesangial cells are susceptible to lipotoxicity, and lipotoxicity-induced apoptosis is involved in DKD [128]. Palmitate-mediated lipotoxicity increased apoptosis and decreased GLP-1R expression in a rat mesangial cell line [129]. Metformin attenuated lipotoxicity-induced mesangial cell apoptosis and restored GLP-1R expression [129], suggesting that GLP-1R activation contributes to an improvement in mesangial cells.

GLP-1RAs improve endothelial dysfunction induced by diabetes [130], helping to prevent the progression of DKD. Improvement in endothelial dysfunction decreases systemic production of inflammatory cytokines and ROS, which may also be reno-protective. GLP-1RAs have been associated with increased mitochondrial biogenesis and improved mitochondrial function [131], thereby reducing systemic ROS production, contributing to renoprotection.

### 3.3. Emerging Therapies for DKD

#### 3.3.1. Dotinurad

Hyperuricemia may be associated with a higher risk of DKD progression [88]. Very recently, a meta-analysis evaluated febuxostat, an xanthine oxidase (XO) inhibitor, for treating DKD with hyperuricemia, showing that febuxostat significantly reduced serum UA, UACR, and serum creatinine levels, and improved eGFR [132].

CKD patients are at increased risk of developing cardiovascular disease due to CKD-specific risk factors [133]. The accumulation of uremic toxins in the circulation and in tissues is associated with the progression of CKD and its co-morbidities, such as cardiovascular disease [133]. ABCG2 is a major transporter of the uremic toxin indoxyl sulfate (IS) [134]. ABCG2 regulates the pathophysiological excretion of IS and strongly influences CKD prognosis. Febuxostat has been reported to inhibit human ABCG2 at a clinical dose [135]. IS accumulates in the body in CKD. An ABCG2 inhibitor, such as febuxostat, causes a marked accumulation of renal IS in rats by suppressing its excretion [136]. Hypouricemic agents that do not affect ABCG2 are effective therapeutic options for the treatment of hyperuricemia in patients with CKD.

**Figure 4:**
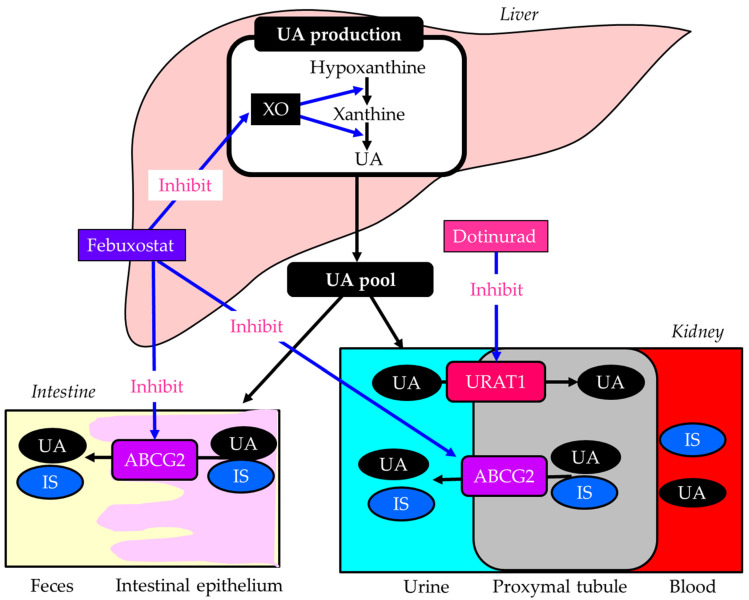
The inhibitory effects of febuxostat and dotinurad on molecules associated with uric acid and uremic toxin indoxyl sulfate metabolism. Black arrows indicate the flow of substance and blue arrows indicate inhibitory effects of febuxostat and dotinurad on molecules associated with uric acid and indoxyl sulfate metabolism. ATP-binding cassette, subfamily G, 2 (ABCG2); IS, indoxyl sulfate; UA, uric acid; URAT1, urate transporter 1; XO, xanthine oxidase.

Dotinurad is a new therapeutic medicine for gout and hyperuricemia discovered by FUJI YAKUHIN, a Japanese pharmaceutical company. The inhibitory effects of febuxostat and dotinurad on molecules associated with UA and uremic toxin IS metabolism were shown in Figure 4. Dotinurad reduces serum UA levels by selectively inhibiting URAT1 [137]. Dotinurad is a potent selective URAT1 inhibitor with minimal effect on other urate transporters, such as ABCG2 [137], indicating that dotinurad reduces serum UA by not affecting IS clearance. Such characteristics of dotinurad are beneficial for DKD. In fact, dotinurad improved UACR and eGFR in patients with DKD/CKD in our study [138]. This study was independent and not funded by the pharmaceutical company that sells dotinurad. Another study showed that the proportion of patients with improved eGFR was significantly higher in patients with eGFR < 30 than in patients with 30 ≤ eGFR, and dotinurad significantly improved eGFR in advanced CKD patients with eGFR < 30 [139]. A recent comparative study of the effects of dotinurad and febuxostat on renal function in CKD patients with hyperuricemia showed that dotinurad and febuxostat decreased serum UA, and eGFR improved only with dotinurad, but no change was observed with febuxostat, thus highlighting the renal protective effect of dotinurad beyond the reduction in serum UA levels [140]. The difference in the effects of dotinurad and febuxostat on eGFR may be due to different effects of these drugs on ABCG2 and the resulting effects on IS clearance. A very recent study evaluated switching from febuxostat to dotinurad and found that urinary IS increased significantly at weeks 4 and 12 and that IS clearance was significantly improved by dotinurad at week 24 [141], supporting our hypothesis. Dotinurad can be a potential therapeutic option for DKD complicated with hyperuricemia.

Dotinurad was first developed and released in Japan, so no studies on dotinurad have been published outside of Japan to date. Therefore, there is a high concentration of Japanese studies on dotinurad, raising the possibility of regional bias. A broader international representation of evidence would strengthen the manuscript. To confirm the renal protective effect of dotinurad, studies examining the effects of dotinurad on renal function are awaited from countries other than Japan.

#### 3.3.2. Imeglimin

**Figure 5:**
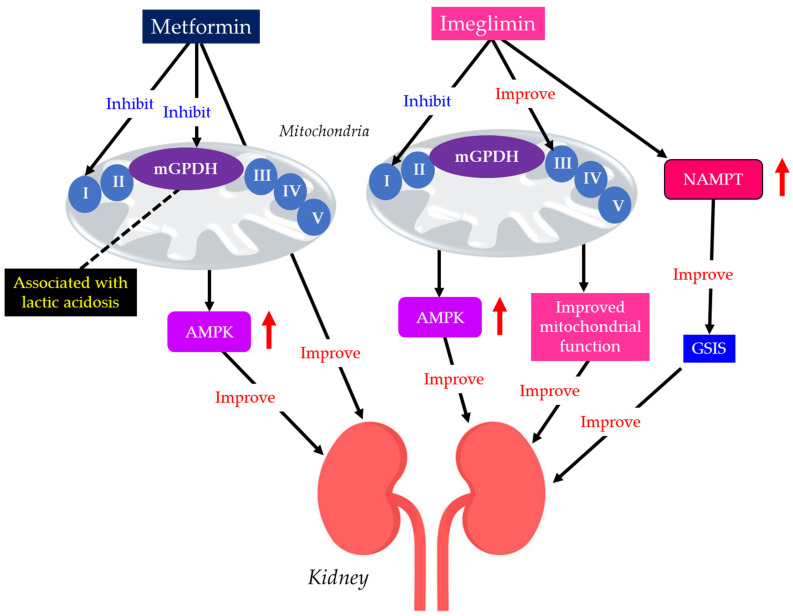
Differential effects of metformin and imeglimin on mitochondria, with resulting differential renal protective effects and risk of lactic acidosis. Black arrows indicate effects and red upward arrows indicate an increase in activity. AMPK, AMP-activated protein kinase; GSIS, glucose-stimulated insulin secretion; mGPDH, mitochondrial glycerol-3-phosphate dehydrogenase; NAMPT, nicotinamide phosphoribosyltransferase.

Imeglimin was discovered by the French company Poxel and developed in Japan by Sumitomo Pharma. It was approved in Japan in 2021, making it the first in the world. Differential effects of metformin and imeglimin on mitochondria, with resulting differential renal protective effects and risk of lactic acidosis were shown in Figure 5. Imeglimin has a chemical structure similar to metformin, but, unlike metformin, it is thought to exert its blood glucose-lowering effect by enhancing insulin secretion [142]. Imeglimin is a novel oral hypoglycemic agent with a unique mechanism of action that targets mitochondrial bioenergetics.

Metformin acts primarily by mildly and reversibly inhibiting mitochondrial complex I in the electron transport chain, especially in hepatocytes. This inhibition reduces ATP production, elevates the AMP/ATP ratio, and activates AMPK, thereby improving insulin resistance [143]. An improvement in insulin resistance reduces hepatic gluconeogenesis and increases glucose uptake in muscles [143].

Recently, a retrospective observational multicenter cohort study including 316,693 patients with type 2 diabetes demonstrated that the metformin group showed a lower incidence of doubling of serum creatinine (HR, 0.71; 95% CI, 0.65–0.77), eGFR ≤ 15 mL/min/1.73 m^2^ (HR 0.61; 95% CI, 0.53–0.71), and ESRD (HR 0.55; 95% CI, 0.47–0.66) [144]. The multivariable Cox survival model showed that metformin users had significantly better renal outcomes, with a notably lower risk of sustained eGFR of <60 mL/min/1.73 m^2^ (HR, 0.71; 95% CI, 0.56–0.90) and new CKD onset (HR, 0.78; 95% CI, 0.65–0.94) [145].

Metformin protects the kidneys mainly via AMPK signaling and AMPK-independent pathways [146]. Metformin exerts multiple mechanisms of renal protection, including induction of autophagy, antioxidant properties, alleviation of ER stress, anti-inflammatory effects, attenuation of lipotoxicity, and antifibrotic effects [146]. Angiotensin, glucose, and oxidative stress induce TGF-β overexpression in tubular epithelial cells, macrophages, and renal interstitial fibroblasts. Metformin attenuated TGF-β expression in renal tissues from a rat model of renal fibrosis [147].

Imeglimin redirects substrate flux towards complex II, inhibits complex I, restores complex III activity in mitochondria, and promotes FA oxidation [142]. Imeglimin also decreases ROS production and improves mitochondrial and ER function. Imeglimin enhances glucose-stimulated insulin secretion (GSIS) by β-cells and inhibits β-cell apoptosis by maintaining mitochondrial and ER function and structure [142].

Pancreatic islet β-cell dysfunction, characterized by defective GSIS, is a predominant component of the pathophysiology of diabetes. Imeglimin acutely and directly amplifies GSIS in pancreatic islets by increasing the cellular nicotinamide adenine dinucleotide (NAD+) through the induction of nicotinamide phosphoribosyltransferase (NAMPT), along with the augmentation of glucose-induced ATP levels [148], which is the crucial difference between imeglimin and metformin.

Some individuals, such as patients with renal impairment, may not be prescribed metformin due to the risk of lactic acidosis [149]. Due to the predominant renal excretion, DKD patients treated with metformin are likely to develop lactic acidosis. A high dose of metformin or imeglimin was administered to rats with renal insufficiency, but only metformin developed fatal lactic acidosis [150]. Only metformin showed an inhibitory effect on the mitochondrial glycerol-3-phosphate dehydrogenase (mGPDH), resulting in a decrease in entry of glycerol into gluconeogenic flux, disrupting the glycerophosphate shuttle and inducing accumulation of cytosolic NADH, which is closely associated with the development of lactic acidosis [151,152], which is also the crucial difference between imeglimin and metformin.

The 90-day imeglimin treatment reduced albuminuria in Zucker rats [153]. This imeglimin treatment significantly reduced renal interstitial fibrosis, glomerular injury, and renal interstitial inflammation. In a retrospective study investigating the safety of imeglimin in patients with an eGFR < 45 mL/min per 1.73 m^2^, no deterioration in renal or hepatic function was observed [154]. Furthermore, significant improvements in proteinuria and acidemia were observed. Another study also found that imeglimin reduced UACR in patients with type 2 diabetes [155].

Imeglimin activates AMPK in the same way as metformin [156]. Improvement of mitochondrial function and reduction of ER stress by imeglimin are thought to result in reduced vascular endothelial dysfunction and improved pathology in DKD [142]. The interaction of insulin with the kidneys is a dynamic, multifocal process, as it acts at multiple sites throughout the nephron [157]. Insulin acts on a range of tissues, from the glomerulus to the renal tubule, by modulating functions such as glomerular filtration, gluconeogenesis, natriuresis, glucose uptake, ion transport regulation, and apoptosis prevention [157]. Therefore, enhancement of GSIS by imgelimin may also contribute to renal protection [148].

Imeglimin was first released in Japan, so no studies on dotinurad have been published outside of Japan to date. Therefore, there is a high concentration of Japanese studies on imeglimin, raising the possibility of regional bias. A broader international representation of evidence would strengthen the manuscript. To confirm the renal protective effect of imeglimin, studies examining the effects of dotinurad on renal function are awaited from countries other than Japan.

#### 3.3.3. Pemafibrate

Hypertriglyceridemia is a common feature of dyslipidemia in DKD and significantly contributes to its progression, acting as an independent predictor of albuminuria and renal function decline, driving renal damage through lipotoxicity, inflammation, and oxidative stress [158,159]. A large population cohort study showed that HDL-C < 40 mg/dL in men and <50 mg/dL in women was associated with a 27% higher risk of low eGFR and a 28% higher risk of an eGFR reduction > 30%, with a 24% higher risk of developing albuminuria and a 44% higher risk of developing one abnormality [160]. Low HDL reduces anti-inflammatory and antioxidant protection, contributing to renal structural damage [161].

**Figure 6:**
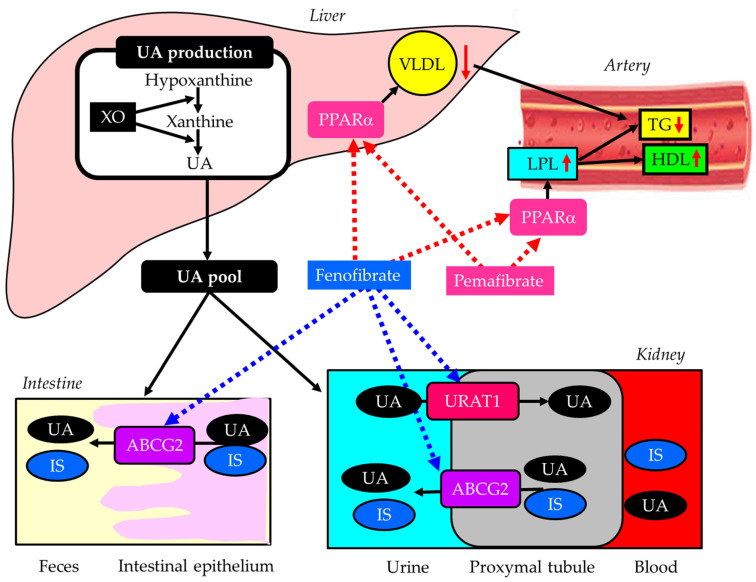
Effects of fenofibrate and pemefibrate on lipid, uric acid, and indoxyl sulfate metabolism. Black horizontal arrows indicate the flow of substance and effects and red upward and downward arrows indicate an increase or decrease in activity or substances, respectively. The red dotted arrows indicate activating effects and the blue dotted arrows indicate inhibitory effects. ATP-binding cassette, subfamily G, 2 (ABCG2); HDL, high-density lipoprotein; IS, indoxyl sulfate; LPL, lipoprotein lipase; PPARα, peroxisome proliferator-activated receptor alpha; TG, triglyceride; UA, uric acid; URAT1, urate transporter 1; VLDL, very-low-density lipoprotein; XO, xanthine oxidase.

Fibrates are effective, first-line medications for managing hypertriglyceridemia and low HDL-C [162]. Effects of fenofibrate and pemefibrate on lipid, UA, and IS metabolism were shown in Figure 6. They act as peroxisome proliferator-activated receptor alpha (PPARα) agonists, increasing lipoprotein lipase (LPL) activity and reducing hepatic very-low-density lipoprotein (VLDL) production [162]. Elevated LPL activity by fibrates also increases serum HDL-C [162]. Fibrates are particularly beneficial for combined high triglyceride (TG) and low HDL-C; therefore, managing such lipid abnormality with fibrates may be a promising option for renal protection. Fenofibrate offers notable reno-protective effects, particularly in DKD. It reduces albuminuria and slows the progression of kidney damage by suppressing inflammatory markers such as TGF-β, reducing oxidative stress, and improving endothelial function [163]. The FIELD study showed that the progression to albuminuria was significantly reduced by fenofibrate compared with placebo [164].

However, fibrates should be prescribed with caution in DKD patients, as impaired urinary excretion of fibrates can lead to serious adverse effects, such as rhabdomyolysis. The review of 76 published cases reported that gemfibrozil was the most frequent agent associated with rhabdomyolysis, followed by bezafibrate, fenofibrate, ciprofibrate, and clofibrate [165]. Twenty-three cases were associated with fibrate monotherapy. Sixteen cases had chronic renal failure before fibrate therapy [165]. Another concern when administering fenofibrate to patients with DKD is its inhibitory effect on ABCG2, a major transporter of IS [134]. Fenofibrate has been shown to inhibit human ABCG2 by over 80% [135]. The accumulation of uremic toxins, such as IS, in the kidney is associated with the progression of DKD [133].

Pemafibrate is a drug for the treatment of dyslipidemia developed by Japanese Kowa Co., Ltd. (Nagoya, Japan) Pemafibrate is a novel, selective PPARα modulator with higher potency and selectivity than conventional fibrates [166]. Pemafibrate showed higher activity and more selectivity for PPARα than fenofibrate in vitro [166]. Additionally, since pemafibrate is excreted into bile, it can be used in patients with renal impairment, unlike conventional fibrates. Pemafibrate significantly attenuated histological tubular injury in FA overload nephropathy and decreased renal FA content and oxidative stress in mice [167]. Another animal study showed that decreases in GFR and increases in serum creatinine were markedly greater after conventional fibrate (fenofibrate, bezafibrate) use than after pemafibrate use, in a unilateral ureteral obstruction (UUO)-induced renal fibrosis model (UUO mice) and an adenine-induced CKD model (CKD mice) [168]. In CKD mice, pemafibrate suppressed the elevation in plasma creatinine and blood urea nitrogen levels, as well as renal fibrosis. Moreover, pemafibrate inhibited the upregulation of MCP-1, interleukin-1β, TNF-α, and interleukin-6 in the kidneys of CKD mice.

Fenofibrate was reported to decrease serum UA levels by increasing urinary excretion, most likely through inhibition of URAT1 [169]. We evaluated the effects of switching from fenofibrate to pemafibrate and found that eGFR significantly increased at 3, 6, and 12 months despite an increase in serum UA levels in patients with type 2 diabetes [170]. This study was independent and not funded by the pharmaceutical company that sells pemafibrate. Another study also found that the switching from fenofibrate to pemafibrate was significantly associated with an elevation in eGFR in diabetic patients with CKD [171].

Pemafibrate was first developed and released in Japan, so no studies on dotinurad have been published outside of Japan to date. Therefore, there is a high concentration of Japanese studies on dotinurad, raising the possibility of regional bias. A broader international representation of evidence would strengthen the manuscript. To confirm the renal protective effect of pemafibrate, studies examining its effects on renal function are awaited from countries outside Japan.

## 4. Limitations of This Review

The evidence presented for the emerging compounds such as dotinurad, imeglimin, and pemafibrate is considerably less robust and, in some instances, relies on the authors’ own studies, which may weaken the critical balance of the analysis. Causality is occasionally inferred from mechanistic associations due to the lack of high-quality studies. The emerging compounds were first developed and launched in Japan, so no studies on these drugs have been published outside Japan to date. Therefore, there is a high concentration of Japanese studies on dotinurad, raising the possibility of regional bias. A broader international representation of evidence would strengthen the manuscript. To confirm the renal protective effect of these emerging compounds, RCTs examining their effects on renal function are awaited from countries outside Japan.

## 5. Conclusions

**Figure 7:**
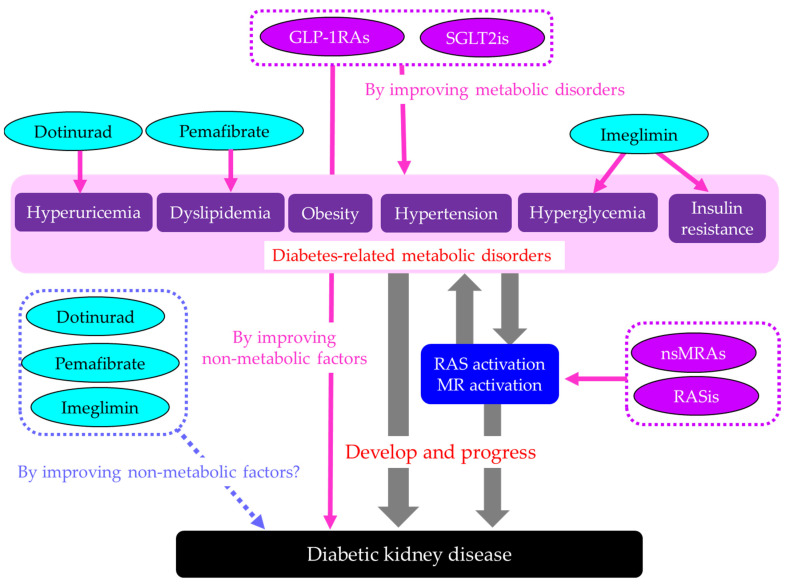
Four pillars and the emerging drugs for DKD. Grey bold arrows indicate pathological associations among metabolic disorders, RAS and MR activation, and DKD. Pink arrows indicate improvement and a dotted blue arrow indicates possible additional beneficial effects of the emerging drugs on DKD. GLP-1RAs, glucagon-like peptide-1 receptor agonists; nsMRAs, non-steroidal mineralocorticoid receptor antagonists; RASis, renin-angiotensin system inhibitors; SGLT2is, sodium-glucose cotransporter 2 inhibitors.

Diabetes-related metabolic disorders such as hyperglycemia, insulin resistance, obesity, hypertension, dyslipidemia, and hyperuricemia are directly associated with the development and progression of DKD and are associated with the activation of RAS and MR, which are also unfavorably associated with DKD and metabolic disorders (Figure 7). GLP-1RAs and SGLT2is may improve DKD by ameliorating multiple metabolic disorders and improving non-metabolic factors. RASis and nsMRAs improve DKD by inhibiting RAS and MR, and these drugs may also break the vicious cycle between metabolic disorders and RAS and MR activation, thereby contributing to DKD improvement. Dotinurad, pemafibrate, and imeglimin improve hyperuricemia, dyslipidemia, hyperglycemia, and insulin resistance, respectively. However, the extent to which emerging therapies improve DKD beyond the effects on metabolic disorders is largely unknown and should be elucidated in the future.

## Data Availability

No new data were created or analyzed in this study. Data sharing is not applicable to this article.

## Abbreviations

ABCG2	ATP-binding cassette, subfamily G, 2
ACEis	angiotensin-converting enzyme inhibitors
AGEs	advanced glycation end products
AMPK	AMP-activated protein kinase
ARBs	AT1R blockers
AT1R	angiotensin II type 1 receptor
BMI	body mass index
CI	confidence interval
CKD	chronic kidney disease
DKD	diabetic kidney disease
ECM	extracellular matrix
eGFR	estimated glomerular filtration rate
ENaC	epithelial sodium channel
eNOS	endothelial nitric oxide synthase
ER	endoplasmic reticulum
ESRD	end-stage renal disease
FA	fatty acid
GBM	glomerular basement membrane
GLP-1RAs	glucagon-like peptide-1 receptor agonists
GLUT9	glucose transporter 9
GSIS	glucose-stimulated insulin secretion
HDL-C	high-density lipoprotein-cholesterol
HR	hazard ratio
IGF-1	insulin-like growth factor 1
IS	indoxyl sulfate
LDL-C	low-density lipoprotein-cholesterol
LPL	lipoprotein lipase
MAMs	mitochondria-associated endoplasmic reticulum membranes
MCP-1	monocyte chemoattractant protein-1
mGPDH	mitochondrial glycerol-3-phosphate dehydrogenase
MR	mineralocorticoid receptor
NAD+	nicotinamide adenine dinucleotide
NAMPT	nicotinamide phosphoribosyltransferase
NF-κB	nuclear factor-κB
NHE3	Na+/H+ exchanger 3
NO	nitric oxide
NLRP3	NOD-like receptor family and pyrin domain-containing 3
nsMRAs	non-steroidal mineralocorticoid receptor antagonists
PI3K-Akt	phosphatidylinositol 3′-kinase-Akt
PKC	protein kinase C
PPARα	peroxisome proliferator-activated receptor alpha
Rac1	Rac family small guanosine triphosphatase 1
RAS	renin-angiotensin system
RASis	RAS inhibitors
RCTs	randomized controlled trials
ROS	reactive oxygen species
SGLT2	sodium-glucose cotransporter 2
SGLT2is	sodium-glucose cotransporter 2 inhibitors
TG	triglyceride
TGF	tubuloglomerular feedback
TGF-β	transforming growth factor beta
TNF-α	tumor necrosis factor-alpha
UA	uric acid
URAT1	urate transporter 1
VEGF	vascular endothelial growth factor
VLDL	very-low-density lipoprotein
XO	xanthine oxidase
